# Intraplexal nerve transfers

**DOI:** 10.1186/1753-6561-9-S3-A27

**Published:** 2015-05-19

**Authors:** Somsak Leechavengvongs

**Affiliations:** 1 Institute of Orthopaedics, Lerdsin General Hospital, Bangkok, 10500, Thailand

## 

Intraplexal nerve transfer is defined as nerve transfer of a nerve within the brachial plexus with intact spinal cord connections to a more important injured nerve. For elbow flexion, the most popular one for the upper arm brachial plexus injury is the “Oberlin” nerve transfer. Transferring a part of the ulnar nerve to the branch to the biceps (Oberlin 1) and possibly transferring a part of the median nerve to the branch to the brachialis (Oberlin 2) provides promising results for elbow flexion without any permanent deficit of the donor nerve function.

In 1998, we published the paper using the Oberlin 1 technique in 32 cases with upper arm brachial plexus injury. Thirty patients had biceps strength of M4 (flexion power ranged from 0.5 to 7 kg) and 1 had biceps strength of M3. All but 1 patient demonstrated signs of recovery of the biceps muscle. No notable impairment of hand function was observed.

For shoulder abduction, most nerve surgeons recommended nerve transfers both to the suprascapular nerve and the axillary nerve. In 2003, we described the nerve transfer to the deltoid muscle using the nerve to the long head of triceps both anatomically and clinically in 7 cases. All patients recovered deltoid power against resistance (M4) at the last follow-up evaluation. Useful functional recovery was achieved in all 7 patients; 5 had excellent recoveries and 2 had good results. The average shoulder abduction was 124 degrees. No notable weakness of elbow extension was observed.

In 2006, we published the paper of combined nerve transfers for C5 and C6 brachial plexus avulsion injury in 15 cases. All 15 patients achieved useful functional recovery. Ten patients experienced excellent recoveries and 5 were classified as having good results. The mean shoulder abduction was 115°. The mean shoulder external rotation was 97°. No patients complained of functional deficit from harvesting of the nerve to the long head of the triceps.

In our experience, 40 % of our patients with upper arm typed brachial plexus injury demonstrated winged scapula from serratus anterior weakness. In 2009, we described the nerve transfer to serratus anterior muscle using the thoracodorsal nerve in C5 and C6 brachial plexus avulsion injury in 5 patients. All patients recovered serratus anterior muscle function. Two patients had no winged scapula, whereas 3 patients had mild winged scapula after the surgery at the last follow-up evaluation. The result was excellent for 2 patients, good for 2 patients, and fair for 1 patient. The averaged shoulder abduction was increased to 134 degrees and external rotation was 124 degree. No notable weakness of shoulder adduction was observed.

For wrist extension in C5, C6 and C7 brachial plexus injury which is difficult to achieve by tendon transfer, we described the nerve transfer using the branch of median nerve to restore the wrist extension. We used Flexor digitorumsuperficialis branch of median nerve transfer to extensor carpi radialis branch of radial nerve. This technique could restore wrist extension in C5, C6 and C7 brachial plexus root avulsion.

